# Epigenetic Control of Plant Cold Responses

**DOI:** 10.3389/fpls.2017.01643

**Published:** 2017-09-21

**Authors:** Aditya Banerjee, Shabir H. Wani, Aryadeep Roychoudhury

**Affiliations:** ^1^Post Graduate Department of Biotechnology, St. Xavier's College-Autonomous Kolkata, India; ^2^Mountain Research Centre for Field Crops, Sher-e-Kashmir University of Agricultural Sciences and Technology of Kashmir Srinagar, India; ^3^Department of Plant Soil and Microbial Sciences, Michigan State University East Lansing, MI, United States

**Keywords:** cold stress, DNA methylation, histone modifications, vernalization genes, cold acclimation, crosstalks

## Cold stress in plants

Higher plants are sedentary organisms which inevitably endure a variety of environmental stresses throughout the life cycle. Abiotic stresses can be atmospheric like cold, heat and UV irradiation; or can also be edaphic like salinity, drought, and heavy metal toxicity (Wani and Gosal, 2011; Surekha et al., 2015). Of all these, cold stress is regarded as a major environmental factor which limits agricultural expansion and crop productivity in hilly terrains (Sanghera et al., 2011). Non-freezing low temperatures deteriorate plant growth physiology by inducing chilling injuries like photosynthesis-associated damages, chlorosis, unregulated apoptosis, loss of membrane fluidity and ultimately wilting (Wani et al., 2016). Depending on the extent of sensitivity among plants, cold stress has been sub-divided into two types. Chilling stress is characterized by 0–15°C, whereas temperatures below 0°C cause freezing stress (Wani et al., 2013). By virtue of cold acclimation and associated alterations at the molecular and biochemical levels, temperate climatic plants exhibit greater ranges of cold tolerance compared to their tropical and sub-tropical counterparts (Yamaguchi-Shinozaki and Shinozaki, 2006). Deciphering the epigenomic landscape in plants exposed to cold conditions is a rapidly developing field (Hu et al., 2011). Intricate research focussing on epigenetic processes during cold stress has led to the identification of molecular targets which can be genetically manipulated to generate cold tolerant lines.

Vernalization is a floral regulatory process preventing precocious flowering during autumn or winter. It gradually promotes flowering competence after prolonged exposures to cold conditions in a species-dependent manner (Kim et al., 2009). Physiologically, vernalization is a “memory response” which is correlated with epigenetic regulation, as observed in the model plant *Arabidopsis thaliana* (Song et al., 2012).

## Epigenetic control of plants during cold acclimation

### Histone modifications during cold acclimation

The expression of epigenetic regulators varies under cold conditions (Banerjee and Roychoudhury, 2017a). Up regulated expression of the histone deacetylases (*HDAC*s) was observed in *Zea mays* during cold acclimation. This resulted in the global deacetylation at the lysine residues on the histone subunits H3 and H4 (Hu et al., 2011). Cold-dependent alternative splicing was observed in the histone demethylase, Jumonji C domain-containing gene, *JMJC5* in *Medicago truncatula* (Shen et al., 2016). Sequencing of the products from the four alternatively spliced RNA isoforms revealed the presence of three premature termination codon-containing variants and a full length protein. Under cold stress, the protein variant having 3′-alternative splice site at the second intron exhibited less sensitivity than the one containing the splice site at the first intron. All the variants were however sensitive to nonsense-mediated decay (Shen et al., 2016). Selective non-silencing of the heterochromatin tandem repeats was reported in *Z. mays* exposed to cold. These plants accumulated non-canonical histone subunit H3, acetylated on the ninth lysine residue (H3K9ac) along with reduced DNA methylation and dimethylation at H3K9 (H3K9me2) in the unsilenced stretches (Hu et al., 2012). Pan (2013) reviewed that the thermosensory responses in *Arabidopsis* were modulated via the interaction of histone variants like H2A.Z with the nucleosomes.

Phosphorylation of the N-terminal tails of histone H3 are post-translational nucleosomal signals manipulating phytocellular stochastics (Nowak and Corces, 2000). Two closely associated Ser/Thr kinases (*At3g03940* and *At5g18190*) crucially phosphorylate histone H3-Thr 3 (H3T3ph) in the pericentromeric/knob regions in *Arabidopsis* to maintain heterochromatin organization and chromosome functions (Wang et al., 2015). *Arabidopsis* plants exposed to cold stress-like osmotic conditions exhibited increased H3T3ph and H3K4me3 at the genomic level with decreased histone H3 occupancy at the pericentromeric/knob regions (Wang et al., 2015). High H3T3ph levels correlated with the transcriptionally active loci of stress-responsive genes (Bej and Basak, 2017).

**Figure 1:**
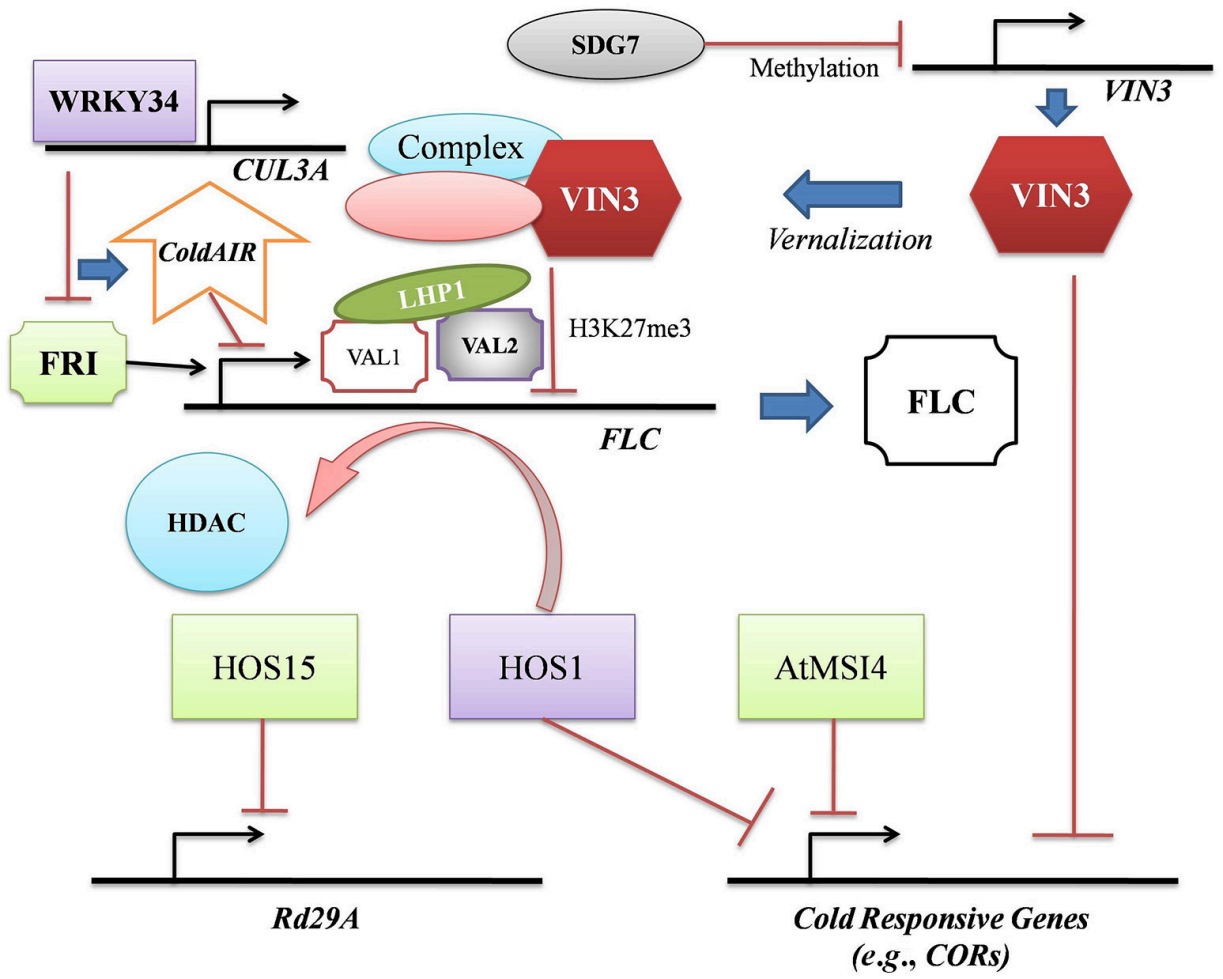
The epigenetic regulation during cold stress highlights that the intricate crosstalks between cold acclimation and vernalization cascades are mediated by VIN3 and HOS1. During vernalization, reduced abundance of SDG7 reverses *VIN3* suppression. Functional VIN3-complex simultaneously inhibits the expression of *COR*s (required for cold acclimation) and *FLC* via repressive histone methylation and promotes vernalization. The epigenetic regulation of *FLC* is multivariate. LHP1 associates with VAL1 and VAL2 to inhibit *FLC* transcription. WRKY34 promotes the expression of *CUL3A*, encoding an ubiquitin E3 ligase. CUL3A proteasomally degrades FRI, the positive regulator of *FLC*. The lncRNA, *ColdAIR* triggers post-transcriptional gene silencing of *FLC* and positively regulates vernalization. The second crosstalk mediator, HOS1 promotes *FLC* expression via HDAC dissociation and also inhibits the transcription of *COR*s. HOS1 thus acts as a negative regulator of vernalization as well as cold acclimation. HOS15 generally improves cold stress tolerance but inhibits the expression of drought-inducible *Rd29A*. MSI4 acts as a negative regulator of cold acclimation.

Cold stress up regulates several downstream transcription factors (TFs) and their target genes (Wani et al., 2016). Histone occupancy-dependent and –independent decrease of H3K27me3 was observed in *Cold Regulated 15A* (*COR15A*) and *Galactinol Synthase 3* in *Arabidopsis*. H3K27me3 decrease permitted the de-repression of these cold responsive genes and ensured systemic acclimatization to low temperatures (Kwon et al., 2009; Banerjee and Roychoudhury, 2016). Gene activating histone acetylation was noted in cold responsive genes like *Drought Responsive Element Binding 1* (*DREB1*) and *COR413* in *Z. mays* exposed to cold (Hu et al., 2011; Banerjee and Roychoudhury, 2017b). Low temperatures induced similar histone acetylation in the *Oryza sativa DREB1b* promoter (Roy et al., 2014). The RING finger E3 ligase, HIGH EXPRESSION OF OSMOTICALLY RESPONSIVE GENE 1 (HOS1) negatively regulates crucial cold responsive genes like *C-repeat binding factors* (*CBF*s)/ *DREB1*s, *COR*s, *Responsive to Dehydration* genes (*RD*s), cold induced genes (*KIN*s), and the cold induced TF, *ICE1* during cold stress (Dong et al., 2006). An interesting epigenetic-floral regulation has been observed in *Arabidopsis* where HOS1 dissociated HDAC6 from the *Flowering Locus C* (*FLC*) chromatin to maintain *FLC* expression (Jung et al., 2013; Figure 1).

Low temperatures induced the accumulation of a WD40-repeat protein, MSI1 (a subunit of the chromatin assembly factor 1) and the 600 kDa polycomb complex (MEDEA, FERTILIZATION-INDEPENDENT ENDOSPERM and FERTILIZATION-INDEPENDENT SEED 2). The MSI-like protein, MSI4/FVE negatively regulated *COR* genes during cold stress in *Arabidopsis* (Dhar et al., 2014; Figure 1). Recent studies have identified distinct RNA recognition motif and PWWP domains in AtMSI4 which regulate the assembly of nucleoprotein complexes and determine the pleiotropic functions in response to stress (Kenzior and Folk, 2015). Significant sequence homology exists between the cold induced WD40-repeat protein, HOS15 and the human transducin-beta like protein 1 (TBL1) (Zhu et al., 2008). TBL1 constitutes the SMRT/N-CoR repressor complex which interacts with HDACs. Thus, deacetylation at H4 is supposedly regulated by HOS15 to improve cold tolerance in *Arabidopsis*. The *hos15* mutant lines exhibited cold hypersensitivity with increased expression of stress-inducible genes like *Rd29A* (Kim et al., 2015) (Figure 1). Both the abscisic acid-dependent and –independent pathways up regulate *Rd29A* expression under drought (Roychoudhury and Banerjee, 2015). Establishment of evidential correlation between HOS15 and H4 deacetylation during *Rd29A* expression can open a new avenue in plant cold tolerance research.

### DNA methylation during cold acclimation

Investigating the spatio-temporal variability in cold-induced DNA methylation is essential since it ensures proper chromatin packaging and conditional gene expression (Banerjee and Roychoudhury, 2017a). Methylation-sensitive amplified fragment-length polymorphism markers detected the changes in cytosine methylation in the alpine subnival plant, *Chorispora bungeana* exposed to 4°C chilling and −4°C freezing stress. Rapid alterations in cytosine methylation occurred throughout the periods of chilling and freezing (Song et al., 2015). Comparative methylome analysis in *Populus simonii* grown under cold, osmotic, heat and salt stresses identified condition-dependent variable cytosine methylation patterns and 1,376 stress-specific differentially methylated regions (SDMRs) (Song et al., 2016). Epigenetic regulation was observed in the SDMR162 region consisting of non-coding RNAs like microRNA 396e (miR396e) and long-non-coding RNA 00268512 (lncRNA00268512). It was proposed that lncRNA00268512 controlled miR396e transcription by acting as a target mimic. Signature methylation marks regulated miR6445a stability in *P. simonii* exposed to cold, salt, osmotic and heat stresses (Song et al., 2016). Non-coding RNAs are also known to play crucial roles in designing the epigenomic landscape in plants exposed to multiple abiotic stresses (Banerjee et al., 2016; Banerjee and Roychoudhury, 2017c). lncRNAs contain conserved stress-specific elements involved in the binding of TFs, phytochrome-interacting factor 4 (PIF4) and PIF5 (Di et al., 2014). Among the 245 poly A+ and 58 poly A- lncRNAs, a salt-responsive UUC motif and a cold-responsive AU-rich stem-loop were annotated (Di et al., 2014). Characterization of such cold stress responsive *cis* acting elements is crucial for deciphering the unidentified transient interactions occurring between the non-coding RNAs and their target genes.

## Vernalization in the context of epigenetics

Vernalization in the *Arabidopsis* ecotypes is regulated by *FLC, Vernalization 1* (*VRN1*), *VRN2* and *Vernalization Insensitive 3* (*VIN3*). Cold stress induces the expression of *VIN3*, a homeodomain finger containing gene encoding protein. VIN3 associates with an unidentified complex and triggers repressive H3K27me3 and H3K9me in the *FLC* chromatin (Kim and Sung, 2017; Figure 1). The complex also initiates loss of H3 acetylation and H3K4 methylation, thus suppressing *FLC* expression and promoting vernalization. It appears that the *VIN3* and *FLC* families actually co-evolved to ensure proper epigenetic regulation of vernalization (Kim and Sung, 2013). In another instance, the H3K27 methyltransferase, SET DOMAIN GROUP 7 (SDG7) negatively regulated the temporal expression of *VIN3* in *Arabidopsis* under control conditions (Figuer 1). Abiding by the *FLC-VIN3* regulatory model, *sdg7 Arabidopsis* mutants exhibited partial vernalization even without cold exposure (Lee et al., 2015). The polycomb group (PcG) proteins along with two homologous *trans*-acting epigenome readers, VIVIPAROUS 1/ABA-INSENSITIVE 3-LIKE 1 (VAL1) and VAL2 stably repress *FLC* during prolonged cold treatment (Yuan et al., 2016). The epigenome readers recognized the *cis*-acting *Cold Memory Elements* and H3K27me3 to directly associate with LIKE HETEROCHROMATIN PROTEIN 1. This increased H3K27me3 in the *FLC* nucleation region leading to its repression during vernalization (Yuan et al., 2016; Figure 1).

The scaffold protein, FRIGIDA (FRI) recruits epigenetic factors to regulate vernalization-related floral gene expression in winter annual accessions of *Arabidopsis* (Hu et al., 2014). It was reported that the TF, WRKY34 promotes the expression of the ubiquitin E3 ligase, *CULLIN 3A* which proteasomally degrades FRI and triggers the accumulation of lncRNA, *Cold Induced Long Antisense Intragenic RNA* (*ColdAIR*) in the late phases of vernalization. High *ColdAIR* levels reduce H3K4me3 in *FLC* and facilitates flowering after vernalization (Hu et al., 2014; Figure 1).

Trans-generational repression of *FLC* during vernalization is maintained as a cellular memory via the mitotic inheritance of the repressive histone marks in the chromatin (Achrem et al., 2012). However, repeated brief cold exposures due to local weather disturbances degrade the central floral regulator, CONSTANS (CO) and delay flowering in *Arabidopsis*. The E3-ubiquitin ligase HOS1 together with its target, CO serve as the molecular integrator of the photoperiodic and cold stress pathways (Jung et al., 2012). Research in temperate cereal crops (*Triticum* spp., *Hordeum vulgare, Avena sativa, Secale cereale* etc.) has verified the increase in gene activating H3K9me3 and decrease in gene suppressing H3K27me3 in *VRN1* during vernalization (Oliver et al., 2009). Such variations in H3K methylation status signify vernalization memory in cereals (Banerjee and Roychoudhury, 2017c). However, increased H3K27me3 and decreased H3K4me3 levels in *VRN2* and *Flowering Locus T1* (*FT1*) were observed in both the vernalized and non-vernalized seedlings. This depicts that unlike in *VRN1*, cold treatment does not modify the chromatin of these genes (Achrem et al., 2012). Vernalization in cereal crops is supposedly mediated by DNA demethylation. The PcG proteins recognizing H3K27me3 sites inhibit *VRN2* and *FT* expression in the germinating caryopses but refrain from regulating *VRN1* expression in the shoot apices (Li and Liu, 2010). Such spatial regulation of epigenetic factors complicates the systemic overview of cold treatment in plants. Vernalized *Arabidopsis* seedlings exposed to 30°C, directly after cold treatment exhibited low H3K27me3 at the *FLC* locus (Bouche et al., 2015). Thus cold shock memory imprinted in the plant epigenome is ushered by the condition-dependent reversible alteration of chromatin architecture.

## Future perspectives

Epigenetic regulation under cold stress is a manifestation of multiple crosstalks (Figure 1) and plant intelligence. Another crosstalk among the genes involved in flowering, vernalization and generation of cold tolerance can be hypothesized in *Triticum monococcum* where VRN1 initiates the regulatory cascade to down-regulate cold acclimation genes like *COR*s and *CBF*s (Galiba et al., 2009). Thus, it is quintessential to design an epigenomic blueprint acting as a global database of chromatin alterations occurring at low temperatures. Molecular characterization of the epigenetic stress memory is crucially required to integrate plant behavioral responses with intelligence. Stress conditions induce the production of volatile organic carbons which often act as inter-species communicating molecules (Ueda et al., 2012). The epigenetic regulation of such metabolite synthesis during cold stress remains to be elucidated. Genome-wide association studies followed by epiallele mining and subsequent annotations on next generation sequencing platforms should be undertaken to unravel the intricate epigenomic landscape especially in staple crops exposed to cold stress. Understanding the spatio-temporal expression of such epialleles, their interactions with small RNAs during cold stress and stringent mapping of candidate quantitative trait loci can identify potential molecular targets which can be engineered to generate tolerant phenotypes. These can then be recommended for field trials.

## Author contributions

AR and SW suggested the title of the opinion article. AB designed the article, drafted the entire manuscript and arranged the references. AR incorporated all the necessary modifications. All the authors checked and confirmed the final version of the manuscript.

### Conflict of interest statement

The authors declare that the research was conducted in the absence of any commercial or financial relationships that could be construed as a potential conflict of interest.
